# Analysis of the current status and associated factors of depressive symptoms in Chinese middle-aged and elderly stroke patients—based on CHARLS data

**DOI:** 10.3389/fpsyt.2025.1602287

**Published:** 2025-06-06

**Authors:** Yage Shi, Chenjun Liu, Xueting Sun, Dingding Li, Shuaiyou Wang, Xinyi Zhu, Kun Pan, Xiaoxia Chen, Huimin Zhang

**Affiliations:** ^1^ School of Nursing, Xinxiang Medical College, Xinxiang, China; ^2^ Nursing Department, Xinxiang First People’s Hospital, Xinxiang, China

**Keywords:** middle-aged and older adults, stroke, depressive symptom, CHARLS, factors

## Abstract

**Background:**

To investigate the prevalence and associated factors of depressive symptoms in middle-aged and elderly stroke patients in China, and to provide reference for improving the mental health of middle-aged and elderly stroke patients in China.

**Methods:**

The data for this study were drawn from the 2020 wave of the China Health and Retirement Longitudinal Study (CHARLS), and middle-aged and elderly stroke patients aged ≥45 years were considered as study subjects (n=988). A simplified version of the Epidemiological Survey Depression Scale score was used to determine depressive symptoms (≥10 points defined as depression) in the study population, and associated factors were analyzed using binary logistic regression.

**Results:**

Among the 988 middle-aged and elderly stroke patients, 547 (55.4%) had depressive symptoms and 441 (44.6%) did not. The results of binary logistic regression analysis showed that education level, history of alcohol consumption, sleep quality, loneliness, self-rated health status, self-rated memory status, life satisfaction, and ADL were the associated factors of depressive symptom in middle-aged and elderly stroke patients (*P* < 0.05).

**Conclusion:**

The study has shown that depressive symptoms in Chinese middle-aged and elderly stroke patients are associated with a variety of factors, and healthcare professionals should regularly assess with early recognition and take interventions to improve the disease.

## Introduction

As the second leading cause of death globally, the post-stroke burden is significant, with studies predicting a 50% increase in stroke mortality—from 66 million in 2020 to 97 million in 2050—and over the same period the disability-adjusted life year (DALY) will increase from 144.8 million in 2020 to 189.3 million in 2050 (1). At the same time, the incidence of stroke is increasing with age, and the number of deaths and disabilities globally is likely to increase significantly in the future. Stroke not only causes complications such as hemiparesis, hemiplegia, aphasia, and ataxia but also leads to emotional and psychiatric disorders in patients with prolonged illness, with post-stroke depression being the most common.

Post-stroke depression (PSD) is one of the most common and serious sequelae of stroke, afflicting approximately one-third of stroke survivors (2), and is the most prevalent neuropsychological disorder among stroke patients, characterized by persistent depressed mood and diminished interest (3). PSD negatively affects the recovery or restoration of motor and cognitive deficits after stroke, and significantly increases the chances of recurrence of neurovascular events (4). Meanwhile, post-stroke depression prevents patients from actively participating in exercise, decreases adherence to rehabilitation exercises, increases stroke recurrence and mortality rates, affects patients’ ability to perform daily living activities, and leads to poor quality of life (5). This brings a heavy burden to today’s society and patients’ families and also has a significant impact on the course, recovery, and prognosis of stroke. The level of mental health of stroke patients seriously affects their functional recovery and cognitive function. Studies have shown that early medication or psychological intervention is beneficial to patients in controlling the progression of the disease (6–8).

Therefore, based on data from the 2020 China Health and Retirement Longitudinal Study (CHARLS), this study systematically explored the prevalence of depressive symptoms and their associated factors in middle-aged and elderly Chinese stroke patients. The study’s results will provide a scientific basis for clinical health professionals to identify high-prevalence populations early and implement precise psychological interventions. These findings will also provide valuable insights for guiding the formulation of evidence-based and targeted mental health prevention strategies. The in-depth analysis of the mental health status of this group and its associated factors will help improve the prognosis of middle-aged and elderly stroke patients and provide practical guidance for improving their overall quality of life.

## Methods

### Study population and design

The data used in this study were sourced from the China Health and Retirement Longitudinal Study (CHARLS) 2020, a nationally representative longitudinal survey that collects high-quality microdata on middle-aged and elderly households and individuals aged 45 years and older in China, to address the challenges associated with China’s aging population. CHARLS initiated its nationally representative baseline survey in 2011, covering 150 county-level units and 450 village-level units across the country. Subsequently, follow-up surveys have been conducted every 2–3 years, with four national follow-ups completed in 2013, 2015, 2018, and 2020, respectively. CHARLS surveyed approximately 10,000 households in China using a probability sampling method, and in 2020 completed 19,395 individual questionnaires. Inclusion criteria for participants in this study included: (1) respondents with reported stroke; (2) age 45 years and older; (3) respondents who provided clear answers to the Center for Epidemiologic Studies Depression Scale (CESD-10); and (4) no omissions on key variables. Exclusion criteria included (1) lack of information about stroke disease, (2) lack of information about depression, and (3) respondents who refused to participate in the survey or were unable to complete the survey. Based on the above criteria, we finally included and analyzed 988 subjects. CHARLS was approved by the Biomedical Ethics Committee of Peking University (IRB00001052-11015) and informed consent forms were signed by all participants who agreed to take part (9).

### Stroke

In the CHARLS 2020 survey, respondents reported stroke events by answering the following questions. (1) Since your last visit, has your stroke condition shown improvement, remained unchanged, or deteriorated? (2) Has a doctor ever told you that you had a stroke? Respondents who answered “yes” to question (1) and question (2) were included in the study.

### Depressive symptom

Participants were assessed using the widely used 10-item Center for Epidemiologic Studies Depression Scale (CESD-10). This scale assesses both depressed and positive mood and consists of 10 items. Participants were asked about their depressed mood and behaviors one week prior, such as whether they felt depressed, lonely, or fearful. Each question on the CESD-10 was assessed on a 4-point scale from 0 to 3 [0 = <1 day (never or rarely), 1 = 1–2 days (some days), 2 = 3–4 days (occasionally), and 3 = 5–7 days (always)]. Before calculating the 10-item total score, items 1, 2, 3, 4, 6, 7, 9, and 10 were positively scored, and items 5 and 8 were negatively scored. Scores on the scale range from 0 to 30, with higher scores indicating more severe depressive symptoms (10). Depressive symptoms were defined as a binary dependent variable (1 = yes, 0 = no) with a total score of ≥10 indicating the presence of depressive symptoms (11). The CESD-10 scale has been reported to have adequate reliability and validity among Chinese community-dwelling older adults (12).

### Covariates

#### Sociodemographic information

Sociodemographic factors included age, gender, education level, marital status, type of residence, smoking history, and drinking history. Age was categorized as 45–59 years, 60–74 years, and ≥75 years. Gender was classified dichotomously as male or female. Educational attainment was categorized into four groups: ‘below primary school,’ ‘primary school,’ ‘junior middle school,’ and ‘high school or above.’ Participants were considered to have a spouse if they were currently married and living with their spouse or if they were married and not living with their spouse temporarily for reasons such as work, or they were considered to have no spouse if they were currently separated, divorced from their spouse, widowed, or never married. The type of residence was categorized as city or rural. History of smoking and alcohol consumption was categorized dichotomously as either yes or no.

#### Health status and functioning

Health status and functional ability were assessed across several domains, such as hearing, vision, self-rated health, self-rated memory, types of chronic diseases, activities of daily living (ADL), instrumental activities of daily living (IADL), nighttime sleep duration, and sleep quality. Hearing and vision conditions were categorized as “problematic” and “not problematic”. In this study, “problematic” is defined as “yes” and “not problematic” as “no”. Self-rated health status and memory were classified into five categories based on participants’ self-reported responses in the questionnaire: excellent, very good, good, fair, and poor. Chronic diseases were diagnosed as one of the 14 common chronic diseases such as hypertension, dyslipidemia, diabetes, cancer, liver disease, kidney disease, heart attack, and emotional problems, which were categorized as the presence of one, the presence of two, or the presence of three or more. Respondents’ self-reported activities of daily living (ADL) in the questionnaire comprised six items: dressing, bathing, eating, transferring in and out of bed, toileting, and controlling urination and defecation. Similarly, instrumental activities of daily living (IADL) included six items: performing household chores, preparing meals, shopping, using the telephone, managing medications, and handling household finances. For analytical purposes, responses indicating ‘no difficulty’ for each item were assigned a score of 1 point, while responses such as ‘difficult but still can be completed,’ ‘difficult,’ ‘need help,’ or ‘unable to complete’ were scored as 0 points. The scores for the six items were summed to generate a total score ranging from 0 to 6, with higher scores indicating better functional ability. The higher the score, the better the activity ability. Sleep quality was assessed using participants’ responses to the statement ‘I don’t sleep well,’ with respondents subsequently stratified into four groups based on the frequency of their reported sleep difficulties during the week. Participants were asked to report their average nightly sleep duration over the past month by responding to the question, ‘In the past month, how many hours of actual sleep did you get each night on average?’ Based on their responses, nighttime sleep duration was classified into three categories: less than 6 hours, 6 to 8 hours, and >8 hours.

#### Environmental factors

Environmental factors include health insurance, pension insurance, employment status, life satisfaction, and social participation. Health insurance, pension insurance, and work status are categorized as “yes” or “no”. Life satisfaction was classified into five levels according to participants’ self-reported responses: extremely satisfied, very satisfied, quite satisfied, not very satisfied, and very dissatisfied. The assessment of social participation was based on participants’ responses to the item, ‘Have you engaged in any of the following social activities in the past month?’ Respondents were stratified into four groups according to their reported level of engagement: no, one type, two types, or three or more types of activities.

### Statistical analysis

Stata/SE 16.0 was used to clean the CHARLS 2020 data. IBM SPSS Statistics 25.0 was used for statistical analysis. For measures that conformed to normal distribution, they were described using mean ± standard deviation, and those that did not conform to normal distribution were described using median and interquartile spacing. Count data were described by frequency and percentage. One-way analysis was performed using a t-test, χ2 test and rank sum test. Variables that were statistically significant in the one-way analysis were included in the binary Logistic regression analysis. The test level was α = 0.05, and *P* < 0.05 indicated that the difference was statistically significant.

## Results

Among 988 middle-aged and elderly stroke patients, 547 (55.4%) exhibited depressive symptoms, while 441 (44.6%) showed no signs of depression. Of the patients with depressive symptoms, 242 (44.2%) were male and 305 (55.8%) were female. In terms of age distribution, 117 (21.4%) were aged 45–59 years, 337 (61.6%) were aged 60–74 years, and 93 (17%) were 75 years or older. Other general data information is shown in **Table 1**.

**Table 1:** General information survey form for study subjects.

Variable	Frequency (N)	Percentage (%)
Age(years)		
45-59	211	21.4
60-74	610	61.7
≥75	167	16.9
Sex		
Female	463	46.9
Male	525	53.1
Type of residence		
City	394	39.9
Rural	594	60.1
Educational level		
Below primary school level	436	44.1
Primary school	238	24.1
Junior middle school	204	20.6
High school or above	110	11.1
Marital status		
Mateless	190	19.2
Has a spouse	798	80.8
Drinking		
No	696	70.4
Yes	292	29.6
Smoking		
No	507	51.3
Yes	481	48.7
Hospitalization insurance		
No	45	4.6
Yes	943	95.4
Endowment insurance		
No	150	15.2
Yes	838	84.8
Vision status		
No	956	96.8
Yes	32	3.2
Hearing status		
No	922	93.3
Yes	66	6.7
Types of chronic diseases		
One kind	45	4.6
Two types	123	12.4
Three or more	820	83
Work status		
No	499	50.5
Yes	489	49.5
Types of social participation		
No	529	53.5
One kind	273	27.6
Two types	136	13.8
Three or more	50	5.1
Hour of sleep		
< 6h	467	47.3
6-8h	443	44.8
> 8h	78	7.9
Sleep quality		
Very good	379	38.4
Good	132	13.4
Bad	191	19.3
Very bad	286	28.9
Aloneness		
Very few	592	59.9
Not many	91	9.2
One half	164	16.6
The majority	141	14.3
Self-rated health		
Very poor	180	18.2
Below average	329	33.3
Average	383	38.8
Fairly good	54	5.5
Very good	42	4.3
Self-rated memory		
Poor	434	43.9
Average	476	48.2
Good	45	4.6
Very good	28	2.8
Exceptional	5	0.5
Life satisfaction		
Very dissatisfied	46	4.7
Not very satisfied	114	11.5
Quite satisfied	501	50.7
Very satisfied	275	27.8
Extremely satisfied	52	5.3
ADL		
0	24	2.4
1	36	3.6
2	47	4.8
3	67	6.8
4	84	8.5
5	179	18.1
6	551	55.8
IADL		
0	25	2.5
1	47	4.8
2	57	5.8
3	83	8.4
4	92	9.3
5	152	15.4
6	532	53.8

### Univariate analysis of the occurrence of depressive symptoms in middle-aged and elderly stroke patients

There was statistical significance between stroke patients with and without depressive symptoms in terms of gender, type of residence, education level, marital status, history of alcohol consumption, history of smoking, type of chronic disease, sleep duration, sleep quality, loneliness, self-assessed health status, self-assessed memory status, life satisfaction, ADL and IADL (*P* < 0.05) (**Table 2**).

**Table 2:** Univariate analysis of depressive symptoms in middle-aged and elderly stroke patients.

Variable	No depression (N=441)	Depression (N=547)	*χ^2^/Z*	*P*
Age (years)			-0.035	0.972
45-59	94 (21.3%)	117 (21.4%)		
60-74	273 (61.9%)	337 (61.6%)		
≥75	74 (16.8%)	93 (17%)		
Sex			38.949	<0.001
Female	158 (35.8%)	305 (55.8%)		
Male	283 (64.2%)	242 (44.2%)		
Type of residence			11.669	<0.001
City	202 (45.8%)	192 (35.1%)		
Rural	239 (54.2%)	355 (64.9%)		
Educational level			-5.007	<0.001
Below primary school level	165 (37.4%)	271 (49.5%)		
Primary school	97 (22%)	141 (25.8%)		
Junior middle school	114 (25.9%)	90 (16.5%)		
High school or above	65 (14.7%)	45 (8.2%)		
Marital status			8.362	0.004
Mateless	67 (15.2%)	123 (22.5%)		
Has a spouse	374 (84.8%)	424 (77.5%)		
Drinking			34.149	<0.001
No	269 (61%)	427 (78.1%)		
Yes	172 (39%)	120 (21.9%)		
Smoking			11.342	<0.001
No	200 (45.4%)	307 (56.1%)		
Yes	241 (54.6%)	240 (43.9%)		
Hospitalization insurance			1.573	0.21
No	16 (3.6%)	29 (5.3%)		
Yes	425 (96.4%)	518 (94.7%)		
Endowment insurance			0.497	0.481
No	63 (14.3%)	87 (15.9%)		
Yes	378 (85.7%)	460 (84.1%)		
Vision status			1.409	0.235
No	430 (97.5%)	526 (96.2%)		
Yes	11 (2.5%)	21 (3.8%)		
Hearing status			0.786	0.375
No	415 (94.1%)	507 (92.7%)		
Yes	26 (5.9%)	40 (7.3%)		
Types of chronic diseases			33.578	<0.001
One kind	29 (6.6%)	16 (2.9%)		
Two types	80 (18.1%)	43 (7.9%)		
Three or more	332 (75.3%)	488 (89.2%)		
Work status			0.538	0.463
No	217 (49.2%)	282 (51.6%)		
Yes	224 (50.8%)	265 (48.4%)		
Types of social participation			5.234	0.155
No	230 (52.2%)	299 (54.7%)		
One kind	119 (27%)	154 (28.2%)		
Two types	62 (14.1%)	74 (13.5%)		
Three or more	30 (6.8%)	20 (3.7%)		
Hour of sleep			-8.015	<0.001
< 6h	141 (32%)	326 (59.6%)		
6-8h	260 (59%)	183 (33.5%)		
> 8h	40 (9.1%)	38 (6.9%)		
Sleep quality			-16.078	<0.001
Very good	281 (63.7%)	98 (17.9%)		
Good	70 (15.9%)	62 (11.3%)		
Bad	46 (10.4%)	145 (26.5%)		
Very bad	44 (10%)	242 (44.2%)		
Aloneness			-17.393	<0.001
Very few	397 (90%)	195 (35.6%)		
Not many	25 (5.7%)	66 (12.1%)		
One half	9 (2%)	155 (28.3%)		
The majority	10 (2.3%)	131 (23.9%)		
Self-rated health			-10.308	<0.001
Very poor	37 (8.4%)	143 (26.1%)		
Below average	115 (26.1%)	214 (39.1%)		
Average	222 (50.3%)	161 (29.4%)		
Fairly good	38 (8.6%)	16 (2.9%)		
Very good	29 (6.6%)	13 (2.4%)		
Self-rated memory			-7.911	<0.001
Poor	133 (30.2%)	301 (55%)		
Average	259 (58.7%)	217 (39.7%)		
Good	27 (6.1%)	18 (3.3%)		
Very good	18 (4.1%)	10 (1.8%)		
Exceptional	4 (0.9%)	1 (0.2%)		
Life satisfaction			-8.365	<0.001
Very dissatisfied	3 (0.7%)	43 (7.9%)		
Not very satisfied	13 (2.9%)	101 (18.5%)		
Quite satisfied	241 (54.6%)	260 (47.5%)		
Very satisfied	154 (34.9%)	121 (22.1%)		
Extremely satisfied	30 (6.8%)	22 (4%)		
ADL			-11.053	<0.001
0	4 (1%)	20 (3.7%)		
1	5 (1.1%)	31 (5.7%)		
2	6 (1.4%)	41 (7.5%)		
3	16 (3.6%)	51 (9.3%)		
4	18 (4.1%)	66 (12%)		
5	65 (14.7%)	114 (20.8%)		
6	327 (74.1%)	224 (41%)		
IADL			-10.719	<0.001
0	2 (0.5%)	23 (4.2%)		
1	11 (2.5%)	36 (6.6%)		
2	12 (2.7%)	45 (8.2%)		
3	19 (4.3%)	64 (11.7%)		
4	19 (4.3%)	73 (13.3%)		
5	62 (14%)	90 (16.5%)		
6	316 (71.7%)	216 (39.5%)		

### Binary logistic regression analysis of the occurrence of depressive symptoms in middle-aged and elderly stroke patients

A binary logistic regression model was constructed, using depression status (present or absent) among middle-aged and elderly stroke patients as the dependent variable and incorporating variables that exhibited statistical significance in the prior univariate analysis as independent variables. The results of the binary logistic regression analysis showed that education level, history of alcohol consumption, sleep quality, loneliness, self-assessed health status, self-assessed memory status, life satisfaction and ADL were the associated factors of depressive symptom in middle-aged and elderly stroke patients (*P* < 0.05) (**Table 3**).

**Table 3:** Binary logistic regression analysis of depressive symptoms in middle-aged and elderly stroke patients.

Variable	β	SE	Wald χ^2^	*P*	OR (95%CI)
Aloneness					
Very few					1
Not many	2.132	0.329	41.945	<0.001	8.434 (4.424-16.08)
One half	3.551	0.405	76.774	<0.001	34.842 (15.746-77.101)
The majority	2.927	0.405	52.265	<0.001	18.667 (8.443-41.274)
Sleep quality					
Very good					1
Good	0.699	0.287	5.95	0.015	2.012 (1.147-3.529)
Bad	2.081	0.279	55.744	<0.001	8.015 (4.641-13.842)
Very bad	2.283	0.265	74.154	<0.001	9.803 (5.831-16.482)
Self-rated health					
Very poor					1
Below average	-0.284	0.312	0.831	0.362	0.753 (0.409-1.386)
Average	-0.803	0.315	6.498	0.011	0.448 (0.242-0.831)
Fairly good	-1.251	0.532	5.523	0.019	0.286 (0.101-0.813)
Very good	-0.919	0.662	1.926	0.165	0.399 (0.109-1.46)
Drinking					
No					1
Yes	-0.522	0.224	5.457	0.019	0.593 (0.383-0.919)
Educational level					
Below primary school level					1
Primary school	0.059	0.252	0.055	0.815	1.061 (0.647-1.738)
Junior middle school	-0.803	0.284	7.994	0.005	0.448 (0.257-0.782)
High school or above	-0.444	0.332	1.787	0.181	0.641 (0.335-1.23)
ADL					
0					1
1	-0.5	0.961	0.271	0.603	0.606 (0.092-3.985)
2	0.052	0.945	0.003	0.956	1.054 (0.165-6.712)
3	-1.251	0.848	2.175	0.14	0.286 (0.054-1.509)
4	-0.624	0.817	0.583	0.445	0.536 (0.108-2.659)
5	-1.488	0.773	3.701	0.054	0.226 (0.05-1.028)
6	-2.2	0.756	8.466	0.004	0.111 (0.025-0.488)
Life satisfaction					
Very dissatisfied					1
Not very satisfied	0.272	0.855	0.101	0.75	1.313 (0.245-7.02)
Quite satisfied	-1.106	0.776	2.032	0.154	0.331 (0.072-1.514)
Very satisfied	-1.124	0.783	2.061	0.151	0.325 (0.07-1.508)
Extremely satisfied	-1.685	0.869	3.763	0.052	0.185 (0.034-1.018)
Self-rated memory					
Poor					1
Average	-0.868	0.215	16.321	<0.001	0.42 (0.276-0.64)
Good	-0.9	0.468	3.688	0.055	0.407 (0.162-1.019)
Very good	-0.598	0.675	0.786	0.375	0.55 (0.146-2.064)
Exceptional	-1.159	1.258	0.848	0.357	0.314 (0.027-3.698)
**Constant**	2.322	1.103	4.427	0.035	10.192 (-)

## Discussion

In this study, the prevalence of depression in stroke patients was 55.4%, which is higher than the prevalence of post-stroke depression in global statistics. Some studies have statistically found that the prevalence of depression in stroke patients ranges from 11% to 41% over 2 years (2). The reasons for the different prevalence rates may include diagnostic criteria, sample size, and ethnic differences (13). The main population in this study was middle-aged and elderly Chinese stroke patients, and the data were obtained from a long-term longitudinal cohort study in China. The differences in prevalence may be related to the different diagnostic criteria for depression in stroke patients. Follow-up studies can adopt standardized diagnostic criteria to ensure consistency and accuracy of diagnosis; and conduct a multidimensional comprehensive analysis by combining relevant examinations, thus improving the reliability and scientificity of the study results. Stroke severity affects post-stroke depression (14). Patients with severe strokes tend to have higher depression scores. However, the CHARLS database lacks indicators to distinguish between stroke severity and type (e.g., hemorrhagic versus ischemic), which may lead to overestimation of prevalence.Finally, the CHARLS database has a limited number of variables. There may be omissions in variable selection. For example, lack of social support and untreated factors may have contributed to higher prevalence (15, 16). We can address this issue by including more variables in future studies.

Our study found a correlation between sleep quality and post-stroke depression, with stroke patients with poor sleep quality more likely to develop depressive symptoms. A meta-analysis showed a prevalence of insomnia in 38.2% of patients after stroke and an association between sleep and post-stroke depression (17). Sleep disorders were also found to be associated with increased odds of post-stroke depression in a national survey based on a US population (18). Sleep status not only contributes to the diagnosis of post-stroke depression but is also an easily observed clinical manifestation (19). As a risk factor for depression, sleep disorders may occur before or after stroke, affecting patients’ functional recovery after stroke and increasing the risk of post-stroke depression through neurobiological mechanisms (20, 21). In addition to this, impaired cerebral hemodynamics and sympathetic hyperactivity due to sleep disorders (e.g., obstructive sleep apnea) lead to poorer neurological and functional recovery, affecting the quality of sleep and increasing the risk rate of cerebrovascular disease recurrence and death (22). Stroke patients are prone to sleep difficulties that affect sleep quality (23), and changes in sleep quality can lead to changes in mental health. One study found that implementing interventions to improve sleep quality reduced depressive symptoms (24), so assessing and improving sleep quality may help in the early identification and reduce the probability of developing depressive symptoms after stroke. Clinical staff should pay adequate attention to sleep quality in stroke patients and provide effective interventions to improve patients’ prognosis and quality of life.

Loneliness is associated with depressive symptoms after stroke. A Canadian longitudinal study of aging also found that stroke victims who often felt lonely were more likely to be depressed than their peers who did not feel lonely as often (25).Huang H et al (26) scholars found that loneliness is a common phenomenon among stroke patients, adversely affecting the recovery of neurological, psychological, and physical functions while increasing the likelihood of stroke recurrence, disability, and even mortality. Loneliness can negatively affect stroke patients who participate in rehabilitation and other daily activities, leading to poor functional outcomes and social isolation (27), further increasing their risk of depression. Patients who do not have a partner or friends at the time of their stroke feel lonely and are prone to develop depressive symptoms; stroke patients who become less lonely and receive more help develop fewer depressive symptoms (28). Living alone is an important risk factor for depression after stroke (29). Living alone increases the likelihood of loneliness among older adults, which is closely linked to diminished well-being. Reduced well-being leads to a higher prevalence of mental health problems, and a clear association between living alone and depressive symptoms has been observed (30). Therefore reducing loneliness and increasing well-being is essential to promote physical and psychological recovery and improve the mental health of stroke patients. Interventions targeting loneliness in stroke patients may reduce post-stroke psychological sequelae and enhance their overall quality of life (31).In clinical practice, it is essential to not only monitor changes in patients’ clinical conditions but also address the impact of loneliness on the psychological well-being of stroke patients, and implement timely and effective interventions to alleviate loneliness and enhance overall quality of life.

Whether or not alcohol is consumed is one of the factors associated with depressive symptoms in stroke patients. A meta-analysis assessing risk factors for post-stroke depression found a correlation between post-stroke depression and alcohol (32). Moderate drinkers have also been previously shown to be a protective factor for depression after stroke (33). Non-drinkers had higher levels of psychological distress and more cases of anxiety and depression than moderate drinkers (34). Moderate drinkers are more likely to have improved mental health; drinking is often associated with social activities and social support is an important factor in alleviating depression, which gives them more opportunities to engage in social activities and be more satisfied with their lives (35). Stroke patients who consume alcohol may receive more emotional support through social activities, which can lead to fewer depressive symptoms. Alcohol consumption may help to relax and relieve stress. For stroke patients, alcohol consumption may help them cope with the psychological stress of the disease to some extent, thus reducing the occurrence of depression, but this does not mean excessive alcohol consumption. Although moderate alcohol consumption may have a positive effect on depression risk in some cases (36), excessive alcohol consumption can have serious negative health consequences, including an increased risk of depression (37).

A significant association exists between educational attainment and the prevalence of depressive symptoms among stroke patients. This is consistent with the findings of a cross-sectional study from China, which noted a significant difference in educational attainment, with high rates of post-stroke depression in patients with low levels of education (13). A study from South Africa noted that low educational level was a negative factor in the occurrence of depression in stroke patients, which may be due to cognitive biases, life stress, and social roles of the patients (38). Individuals with a high educational background are more likely to be aware of knowledge related to their condition and utilize relevant health education resources in order to improve their health literacy and reduce the development of negative emotions such as anxiety and depression. In contrast, patients with lower levels of education tend to have less knowledge about stroke and its consequences, lack the knowledge to cope with it, and ignore changes in symptoms and functioning, failing to intervene promptly in the progression of complications, which ultimately leads to more serious health consequences (39). Less educated patients tend to have limited social resources, less emotional, financial, and medical support, greater isolation and stress, and are prone to depression (2). Therefore, less educated patients are also at higher risk of developing post-stroke depression or developing depressive symptoms (40, 41). During the treatment period, healthcare professionals should provide tailored health education to stroke patients with lower educational attainment, utilizing clear and accessible language to explain disease-related knowledge. This approach can enhance patients’ understanding of their condition, alleviate fear and misconceptions, and improve their ability to cope with the disease.

Poor self-reported health status is strongly associated with an increased prevalence of depression among stroke patients. A Brazilian study showed that stroke patients who rated their self-rated health as good had fewer depressive symptoms than those who rated their self-rated health as poor (42). Individuals who report poor self-rated health are more likely to develop negative cognitions regarding their physical functioning. Such negative self-perceptions can contribute to emotional distress, heightened concerns about future health outcomes, and exacerbate depressive symptoms (43). It is essential to assist patients in identifying and modifying negative cognitive patterns, fostering the development of positive thought processes, and ultimately improving their emotional well-being. Studies have shown that there is a correlation between depression scores and physical functional status at 6 months after stroke, with depression scores significantly higher in patients with poor physical health than in patients with good health (44). Stroke has a high rate of disability, and when patients’ physical health is affected, the quality of life cannot be ensured, individual self-assessed health will decline, and patients who are physically and mentally traumatized are prone to adverse emotions. High depressive symptom trajectories in stroke patients are associated with severe disability, and patients with severe disability are more likely to be in the moderate and high depressive symptom groups (45). Rehabilitation should be undertaken as early as possible after a stroke to improve the patient’s health and reduce disability, thereby reducing the prevalence of depressive symptoms. In addition, this study found that the memory status of stroke patients also affects their ability to develop depressive symptoms. Acute stroke patients are often accompanied by cognitive dysfunction (46), and this neurocognitive impairment may be a prominent feature of acute stroke, which is mainly manifested by impaired cognitive function such as memory loss and inattention. This cognitive dysfunction not only affects the neurological recovery of patients but also has a significant correlation with the occurrence of post-stroke depression (19). Patients with cognitive impairment after stroke are prone to depression (47, 48). Clinical staff should perform a comprehensive assessment of patients’ cognitive function and implement targeted cognitive training interventions for those with identified impairments. This enhances the memory and cognitive level of stroke patients and increases self-confidence, reducing depressive symptoms.

The relationship between life satisfaction and post-stroke depression was negatively correlated. This is consistent with the findings of a study that specifically examined the factors influencing the depressive status of middle-aged and elderly patients with stroke, heart disease, and other chronic diseases in China, which showed that depression was negatively correlated with subjective satisfaction, and in particular, with life satisfaction (49). Stroke may lead to functional impairments such as limb paralysis, speech disorders, and dysphagia, and patients may feel a reduced quality of life and dissatisfaction with their current life status due to the loss or limitation of physical function (35), which in turn leads to depression. Poor stroke outcomes are associated with poorer quality of life and affect stroke recovery (31). Life satisfaction has been shown to be a negative predictor of depressive symptoms, and interventions to improve life satisfaction can be effective in reducing or preventing depressive symptoms (50). Rehabilitation training, such as physical therapy or speech therapy, can be performed to help stroke patients restore or improve their physical function, increase life satisfaction, and improve their mental health.

There is a negative correlation between ADL scores and depressive symptoms in stroke patients. This is consistent with the results of a prospective study that showed that reduced ADL tend to contribute to the development of depression after stroke (51). After a stroke, patients’ ability to perform their own activities decreases, and they may also need help with basic activities of daily living (ADL), which leads to changes in their standard of living (52), negative psychological emotions such as guilt towards family and friends (53), and ultimately to the development of depression or worsening of their condition. A longitudinal study lasting six years also found that post-stroke depressive symptoms were significantly associated with functional limitations in activities of daily living (54). Patients with poor activities of daily living (ADL) functioning develop chronic stressful stimuli, which may also be an influential factor in the emergence of depression (19). Poor activities of daily living (ADL) may prevent stroke patients from actively participating in rehabilitation and having a better life experience, which may have a profound impact on the recovery of stroke patients. Therefore, family members and clinical staff should also pay attention to patients with severe limitations or low activity, provide them with psychological comfort (55), and guide them to adapt to the change of activity ability caused by the disease and carry out appropriate activities according to their conditions, to improve the depressive symptoms of patients with stroke by improving their ability to perform activities of daily living.

There are some limitations to this study. Firstly, The study utilized a self-report survey, and the diagnosis of stroke relied on self-reports provided by the subjects or other informants, which may have some bias in the diagnosis of stroke. Secondly, although the CESD-10 questionnaire is widely used in today’s surveys, it is not representative of studies of people who are ultimately diagnosed with depression. This is because it measures the respondent’s mental state in the past week, which primarily reflects depressive symptoms, and there may still be a diagnostic difference between that and actual depression. Further research is therefore needed to determine the actual clinical level of depression. Finally, this study was a cross-sectional investigation and did not include an analysis of longitudinal changes over time, making it difficult to confirm causality. Therefore, further longitudinal studies are necessary in the future.

## Conclusion

This study took middle-aged and elderly stroke patients as research subjects to investigate the prevalence of current depressive symptoms and related factors and found that several factors, such as education level, history of alcohol consumption, sleep quality, loneliness, self-assessed health status, self-assessed memory status, life satisfaction, and ADL were correlated with depressive symptoms. Analyzing the associated factors enables healthcare professionals to more accurately evaluate disease susceptibility and design targeted interventions for the treatment and management of middle-aged and elderly stroke patients. This can effectively reduce the development of depressive symptoms in stroke patients and improve their quality of life and recovery process. Future care strategies should prioritize not only the physical rehabilitation of stroke patients but also their psychological well-being, aiming to provide comprehensive support that enhances their overall quality of life.

## Data Availability

Publicly available datasets were analyzed in this study. This data can be found here: http://charls.pku.edu.cn/en/.

## References

[1] Feigin VL Owolabi MO. Pragmatic solutions to reduce the global burden of stroke: a World Stroke Organization-Lancet Neurology Commission. Lancet Neurol. (2023) 22:1160–206. doi: 10.1016/S1474-4422(23)00277-6 PMC10715732 37827183

[2] Guo J Wang J Sun W Liu X. The advances of post-stroke depression: 2021 update. J Neurol. (2022) 269:1236–49. doi: 10.1007/s00415-021-10597-4 34052887

[3] Zhou H Wei YJ Xie GY. Research progress on post-stroke depression. Exp Neurol. (2024) 373:114660. doi: 10.1016/j.expneurol.2023.114660 38141804

[4] Das J G K R. Post stroke depression: The sequelae of cerebral stroke. Neurosci Biobehav Rev. (2018) 90:104–14. doi: 10.1016/j.neubiorev.2018.04.005 29656030

[5] Gnanaprakasam A Solomon JM Roy AK Deshmukh AS Karthikbabu S. Association between depression and adherence to upper limb exercises among community-dwelling stroke survivors: A cross-sectional study. Health Sci Rep. (2024) 7:e70133. doi: 10.1002/hsr2.70133 39435034 PMC11491541

[6] Fang Y Mpofu E Athanasou J. Reducing depressive or anxiety symptoms in post-stroke patients: Pilot trial of a constructive integrative psychosocial intervention. Int J Health Sci (Qassim). (2017) 11:53–8. PMC5654182 29085269

[7] Woranush W Moskopp ML Sedghi A Stuckart I Noll T Barlinn K. Preventive approaches for post-stroke depression: where do we stand? A systematic review. Neuropsychiatr Dis Treat. (2021) 17:3359–77. doi: 10.2147/NDT.S337865 PMC8610752 34824532

[8] Mikami K Jorge RE Moser DJ Arndt S Jang M Solodkin A. Prevention of post-stroke generalized anxiety disorder, using escitalopram or problem-solving therapy. J Neuropsychiatry Clin Neurosci. (2014) 26:323–8. doi: 10.1176/appi.neuropsych.11020047 24457590

[9] Zhao Y Hu Y Smith JP Strauss J Yang G. Cohort profile: the China health and retirement longitudinal study (CHARLS). Int J Epidemiol. (2014) 43:61–8. doi: 10.1093/ije/dys203 PMC3937970 23243115

[10] Rong J Zhang N Wang Y Cheng P Zhao D. Development and validation of a nomogram to predict the depressive symptoms among older adults: A national survey in China. J Affect Disord. (2024) 361:367–75. doi: 10.1016/j.jad.2024.06.036 38897299

[11] Zhou L Ma X Wang W. Relationship between cognitive performance and depressive symptoms in chinese older adults: the China health and retirement longitudinal study (CHARLS). J Affect Disord. (2021) 281:454–8. doi: 10.1016/j.jad.2020.12.059 33360747

[12] Chen H Mui AC. Factorial validity of the Center for Epidemiologic Studies Depression Scale short form in older population in China. Int Psychogeriatr. (2014) 26:49–57. doi: 10.1017/S1041610213001701 24125553

[13] Hu QY Chen YJ Liu J Zhao XP Feng WY Tian JB. A cross-sectional study on post-stroke depression and the quality of life. BMC Psychol. (2024) 12:646. doi: 10.1186/s40359-024-02143-4 39533419 PMC11555978

[14] Chau JPC Lo SHS Zhao J Choi KC Lam SKY Butt L. Factors associated with post-stroke depression in chinese stroke survivors. J Stroke Cerebrovasc Dis. (2021) 30:106076. doi: 10.1016/j.jstrokecerebrovasdis.2021.106076 34507255

[15] Kwon B Lee EJ Park S Lee JS Lee MH Jeong D. Long-term changes in post-stroke depression, emotional incontinence, and anger. J Stroke. (2021) 23:263–72. doi: 10.5853/jos.2020.04637 PMC8189860 34102761

[16] Volz M Möbus J Letsch C. The influence of early depressive symptoms, social support and decreasing self-efficacy on depression 6 months post-stroke. J Affect Disord. (2016) 206:252–5. doi: 10.1016/j.jad.2016.07.041 27513631

[17] Baylan S Griffiths S Grant N Broomfield NM Evans JJ Gardani M. Incidence and prevalence of post-stroke insomnia: A systematic review and meta-analysis. Sleep Med Rev. (2020) 49:101222. doi: 10.1016/j.smrv.2019.101222 31739180

[18] Chen W Shen Y Song S Li X. Association of sleep duration and sleep disorders with post-stroke depression and all-cause and cardiovascular disease mortality in US stroke survivors: results from NHANES 2005–2018. Eur J Med Res. (2025) 30:2. doi: 10.1186/s40001-024-02227-2 39754244 PMC11697810

[19] Liao W Chen D Wu J Liu K Feng J Li H. Risk factors for post-stroke depression in patients with mild and moderate strokes. Med (Baltimore). (2023) 102:e34157. doi: 10.1097/MD.0000000000034157 PMC10313280 37390261

[20] Dong L Brown DL Chervin RD Case E Morgenstern LB Lisabeth LD. Pre-stroke sleep duration and post-stroke depression. Sleep Med. (2021) 77:325–9. doi: 10.1016/j.sleep.2020.04.025 PMC7667889 32828696

[21] Liu F Yang Y Wang S Zhang XL Wang AX Liao XL. Impact of sleep duration on depression and anxiety after acute ischemic stroke. Front Neurol. (2021) 12:630638. doi: 10.3389/fneur.2021.630638 33841304 PMC8032928

[22] Hale E Gottlieb E Usseglio J Shechter A. Post-stroke sleep disturbance and recurrent cardiovascular and cerebrovascular events: A systematic review and meta-analysis. Sleep Med. (2023) 104:29–41. doi: 10.1016/j.sleep.2023.02.019 36889030 PMC10098455

[23] Tan XM Liao ZX Zhao YY Sun XC Yi FL. Changes in depressive symptoms before and after the first stroke: A longitudinal study from China Family Panel Study (CFPS). J Affect Disord. (2023) 340:567–74. doi: 10.1016/j.jad.2023.08.058 37573890

[24] Scott AJ Webb TL Martyn-St James M Rowse G Weich S. Improving sleep quality leads to better mental health: A meta-analysis of randomised controlled trials. Sleep Med Rev. (2021) 60:101556. doi: 10.1016/j.smrv.2021.101556 34607184 PMC8651630

[25] MacNeil A Li G Gulati I Taunque A Jiang Y de Groh M. Depression during the COVID-19 pandemic among older adults with stroke history: Findings from the Canadian Longitudinal Study on Aging. Int J Geriatr Psychiatry. (2024) 39:e6062. doi: 10.1002/gps.v39.2 38380892

[26] Huang H Zhang L Dong W Tu L Tang H Liu S. Stigma and loneliness among young and middle-aged stroke survivors: A moderated mediation model of interpersonal sensitivity and resilience. J Psychiatr Ment Health Nurs. (2024) 31:596–606. doi: 10.1111/jpm.13016 38164762

[27] Shi Y Fong MWM Metts CL LaVela SL Bombardier C Hu L. Dynamics of perceived social isolation, secondary conditions, and daily activity patterns among individuals with stroke: A network analysis of ecological momentary assessment data. Arch Phys Med Rehabil. (2024) 105:1314–21. doi: 10.1016/j.apmr.2024.02.733 PMC11227394 38458373

[28] Elayoubi J Haley WE Nelson ME Hueluer G. How social connection and engagement relate to functional limitations and depressive symptoms outcomes after stroke. Stroke. (2023) 54:1830–8. doi: 10.1161/STROKEAHA.122.042386 PMC10313154 37363947

[29] Wang Z Zhu M Su Z Guan B Wang A Wang Y. Post-stroke depression: different characteristics based on follow-up stage and gender-a cohort perspective study from Mainland China. Neurol Res. (2017) 39:996–1005. doi: 10.1080/01616412.2017.1364514 28828931

[30] Xiao A Wang R Liu C Wang X. Influencing factors and predictive models of early post-stroke depression in patients with acute ischemic stroke. BMC Neurol. (2025) 25:104. doi: 10.1186/s12883-025-04090-y 40075309 PMC11900648

[31] Theeke L Horstman P Mallow J Lucke-Wold N Culp S Domico J. Quality of life and loneliness in stroke survivors living in Appalachia. J Neurosci Nurs. (2014) 46:E3–15. doi: 10.1097/JNN.0000000000000097 PMC4413920 25365057

[32] Perrain R Mekaoui L Calvet D Mas JL Gorwood P. A meta-analysis of poststroke depression risk factors comparing depressive-related factors versus others. Int Psychogeriatr. (2020) 32:1331–44. doi: 10.1017/S1041610219002187 32014074

[33] Qiu X Miao J Lan Y Sun W Chen Y Cao Z. Association of cerebral artery stenosis with post-stroke depression at discharge and 3 months after ischemic stroke onset. Front Psychiatry. (2020) 11:585201. doi: 10.3389/fpsyt.2020.585201 33324257 PMC7723904

[34] Rodgers B Parslow R Degenhardt L. Affective disorders, anxiety disorders and psychological distress in non-drinkers. J Affect Disord. (2007) 99:165–72. doi: 10.1016/j.jad.2006.09.006 17046068

[35] Liu Y Liu J Zhou S Xu X Cheng Y Yi Y. Life satisfaction and its influencing factors of middle-aged and elderly stroke patients in China: a national cross-sectional survey. BMJ Open. (2022) 12:e059663. doi: 10.1136/bmjopen-2021-059663 PMC9352989 35922110

[36] Paulson D Shah M Herring D Scott R Herrera M Brush D. The relationship between moderate alcohol consumption, depressive symptomatology, and C-reactive protein: the Health and Retirement Study. Int J Geriatr Psychiatry. (2018) 33:316–24. doi: 10.1002/gps.v33.2 28612359

[37] Gea A Beunza JJ Estruch R Sánchez-Villegas A Salas-Salvadó J Buil-Cosiales P. Alcohol intake, wine consumption and the development of depression: the PREDIMED study. BMC Med. (2013) 11:192. doi: 10.1186/1741-7015-11-192 23988010 PMC3765610

[38] Ojagbemi A Akpa O Elugbadebo F Owolabi M Ovbiagele B. Depression after stroke in sub-saharan africa: A systematic review and meta-analysis. Behav Neurol. (2017) 2017:4160259. doi: 10.1155/2017/4160259 28819339 PMC5551463

[39] Huang Y Wang Q Zou P He G Zeng Y Yang J. Prevalence and factors influencing cognitive impairment among the older adult stroke survivors: a cross-sectional study. Front Public Health. (2023) 11:1254126. doi: 10.3389/fpubh.2023.1254126 37790718 PMC10542404

[40] Backhouse EV Mchutchison CA Cvoro V Shenkin SD Wardlaw JM. Cognitive ability, education and socioeconomic status in childhood and risk of post-stroke depression in later life: A systematic review and meta-analysis. PloS One. (2018) 13:e0200525. doi: 10.1371/journal.pone.0200525 30011299 PMC6047794

[41] Hirata S Ovbiagele B Markovic D Towfighi A. Key factors associated with major depression in a national sample of stroke survivors. J Stroke Cerebrovasc Dis. (2016) 25:1090–5. doi: 10.1016/j.jstrokecerebrovasdis.2015.12.042 26875019

[42] Viana RT de Freitas Araújo É Lima LAO Teixeira-Salmela LF de Morais Faria CDC. General and comparative self-rated health in chronic stroke: an important outcome measure for health professionals. BMC Neurol. (2022) 22:78. doi: 10.1186/s12883-022-02592-7 35255837 PMC8900340

[43] Cai Q Qian M Chen M. Association between socioeconomic status and post-stroke depression in middle-aged and older adults: results from the China health and retirement longitudinal study. BMC Public Health. (2024) 24:1007. doi: 10.1186/s12889-024-18503-z 38605383 PMC11010318

[44] Hu F Zhang K Zhou L Wang Y. The impact of post-stroke depression and physical fatigue on functional status. Actas Esp Psiquiatr. (2025) 53:315–23. doi: 10.62641/aep.v53i2.1688 PMC11898255 40071376

[45] Liu L Li X Marshall IJ Bhalla A Wang Y O'Connell MDL. Trajectories of depressive symptoms 10 years after stroke and associated risk factors: a prospective cohort study. Lancet. (2023) 402 Suppl 1:S64. doi: 10.1016/S0140-6736(23)02111-6 37997108

[46] Girgenti SG Brunson AO Marsh EB. Baseline function and rehabilitation are as important as stroke severity as long-term predictors of cognitive performance post-stroke. Am J Phys Med Rehabil. (2023) 102:S43–s50. doi: 10.1097/PHM.0000000000002125 36634330 PMC11025529

[47] Ojagbemi A Akinyemi R Baiyewu O. Cognitive dysfunction and functional limitations are associated with major depression in stroke survivors attending rehabilitation in Nigeria. NeuroRehabilitation. (2014) 34:455–61. doi: 10.3233/NRE-141061 24463232

[48] Olibamoyo O Adewuya A Ola B Coker O Atilola O. Prevalence and correlates of depression among Nigerian stroke survivors. S Afr J Psychiatr. (2019) 25:1252. doi: 10.4102/sajpsychiatry.v25i0.1252 31205780 PMC6556991

[49] Lin W Zhang D Wang Y Zhang L Yang J. Analysis of depression status and influencing factors in middle-aged and elderly patients with chronic diseases. Front Psychol. (2024) 15:1308397. doi: 10.3389/fpsyg.2024.1308397 38434947 PMC10904536

[50] Liao M Zhang X Xie Z Li L Zou L. The mediating effect of life satisfaction between daily living abilities and depressive symptoms in the Chinese older people: evidence from CHARLS 2020. Front Public Health. (2024) 12:1393530. doi: 10.3389/fpubh.2024.1393530 39211904 PMC11357936

[51] Vojtikiv-Samoilovska D Arsovska A. Prevalence and predictors of depression after stroke - results from a prospective study. Open Access Maced J Med Sci. (2018) 6:824–8. doi: 10.3889/oamjms.2018.182 PMC5985895 29875853

[52] Li J Yang L Lv R Kuang J Zhou K Xu M. Mediating effect of post-stroke depression between activities of daily living and health-related quality of life: meta-analytic structural equation modeling. Qual Life Res. (2023) 32:331–8. doi: 10.1007/s11136-022-03225-9 35972616

[53] Feng Z Li Q Zhou L Chen Z Yin W. The relationship between depressive symptoms and activity of daily living disability among the elderly: results from the China Health and Retirement Longitudinal Study (CHARLS). Public Health. (2021) 198:75–81. doi: 10.1016/j.puhe.2021.06.023 34365109

[54] Ytterberg C Cegrell L Von Koch L Wiklander M. Depression symptoms 6 years after stroke are associated with higher perceived impact of stroke, limitations in ADL and restricted participation. Sci Rep. (2022) 12:7816. doi: 10.1038/s41598-022-11097-9 35551206 PMC9098872

[55] Salter K Foley N Teasell R. Social support interventions and mood status post stroke: a review. Int J Nurs Stud. (2010) 47:616–25. doi: 10.1016/j.ijnurstu.2009.12.002 20053402

