# Correction: Analysis of the current status and associated factors of depressive symptoms in Chinese middle-aged and elderly stroke patients—based on CHARLS data

**DOI:** 10.3389/fpsyt.2025.1647228

**Published:** 2025-07-02

**Authors:** Yage Shi, Chenjun Liu, Xueting Sun, Dingding Li, Shuaiyou Wang, Xinyi Zhu, Kun Pan, Xiaoxia Chen, Huimin Zhang

**Affiliations:** ^1^ School of Nursing, Xinxiang Medical College, Xinxiang, China; ^2^ Nursing Department, Xinxiang First People’s Hospital, Xinxiang, China

**Keywords:** middle-aged and older adults, stroke, depressive symptom, CHARLS, factors

In the published article, there was an error in the **Funding** statement. Only one funder should have been declared. The statement previously said:

“The author(s) declare that financial support was received for the research and/or publication of this article. This work was supported by the 2023 Research and Practice Program on Higher Education Teaching Reform in Henan Province (Graduate Education Category) (grant number2023SJGLX236Y); Henan Province Postgraduate Education Reform and Quality Enhancement Project in 2024 (grant number YJS2024JC27); Henan Province Postgraduate Education Reform and Quality Enhancement Project in 2025 (grant number YJS2025AL91); Philosophy and Social Science Planning Project of Henan Province in 2024 (grant number 2024BSH033); and the Xinxiang Medical College Education Teaching Reform Research and Practice Project (grant number 2024-XYJG-88).”

The corrected **Funding** statement appears below:

“The author(s) declare that financial support was received for the research and/or publication of this article. This work was supported by the Philosophy and Social Science Planning Project of Henan Province in 2024 (grant number 2024BSH033).”

The authors apologize for this error and state that this does not change the scientific conclusions of the article in any way. The original article has been updated.

